# Considerations regarding an updated meta-analysis of the optimal duration of oxaliplatin-based adjuvant chemotherapy in patients with different risk factors for stage II-III colon cancer

**DOI:** 10.1097/JS9.0000000000001350

**Published:** 2024-03-25

**Authors:** Huayang Pang, Menghua Yan, Lihui Chen, Zhou Zhao, Wei Li, Shouru Zhang, Hao Sun

**Affiliations:** a Department of Gastrointestinal Cancer Center, Chongqing University Cancer Hospital; b Chongqing Key Laboratory of Translational Research for Cancer Metastasis and Individualized Treatment, Chongqing University Cancer Hospital, Chongqing, People’s Republic of China

*Dear Editor,*

We have read the recent study by Kuang *et al*.^1^ with great interest. This meta-analysis, which includes six large-scale randomized controlled trials involving 10 332 patients, provides compelling evidence that the 6-month FOLFOX regimen should be exclusively recommended for high-risk stage III colon cancer. Moreover, a 3-month CAPEOX regimen is suggested for stage II–III colon cancer. The authors deserve recognition for offering updated evidence to address the contentious aspects of the 2023 NCCN guidelines. However, there are several concerns that necessitate further clarification.

Firstly, when describing the impact of CAPEOX and FOLFOX on disease-free survival (DFS) in patients with stage II–III colon cancer presenting different risk factors, we observed an inaccurate representation of the total number of patients from the enrolled studies (e.g. 1629 patients in the high-risk II stage subgroup, 3301 patients in the low-risk III stage subgroup, and 4416 patients in the high-risk III stage subgroup). This could potentially mislead readers into believing that these subgroup analyses were based on substantial sample sizes. Therefore, we strongly recommend that the authors provide detailed patient numbers for each subgroup analysis.

Secondly, in Figure 3^1^, we observed that the HORG^2^ data (HR=1.29 [1.08–1.54]) were replicated in both the overall stage II/III group and the high-risk stage III subgroup. However, upon meticulous examination of the primary literature, it is regrettable to note that no data regarding high-risk stage III patients was provided by the HORG trial^2^. After excluding this incorrect data, we found that high-risk stage III patients who underwent a 6-month FOLFOX still exhibited superior DFS compared to those who received a 3-month FOLFOX (HR=1.37 [1.11–1.69]; *I*^2^=2%; Fig. 1A).

**Figure 1:**
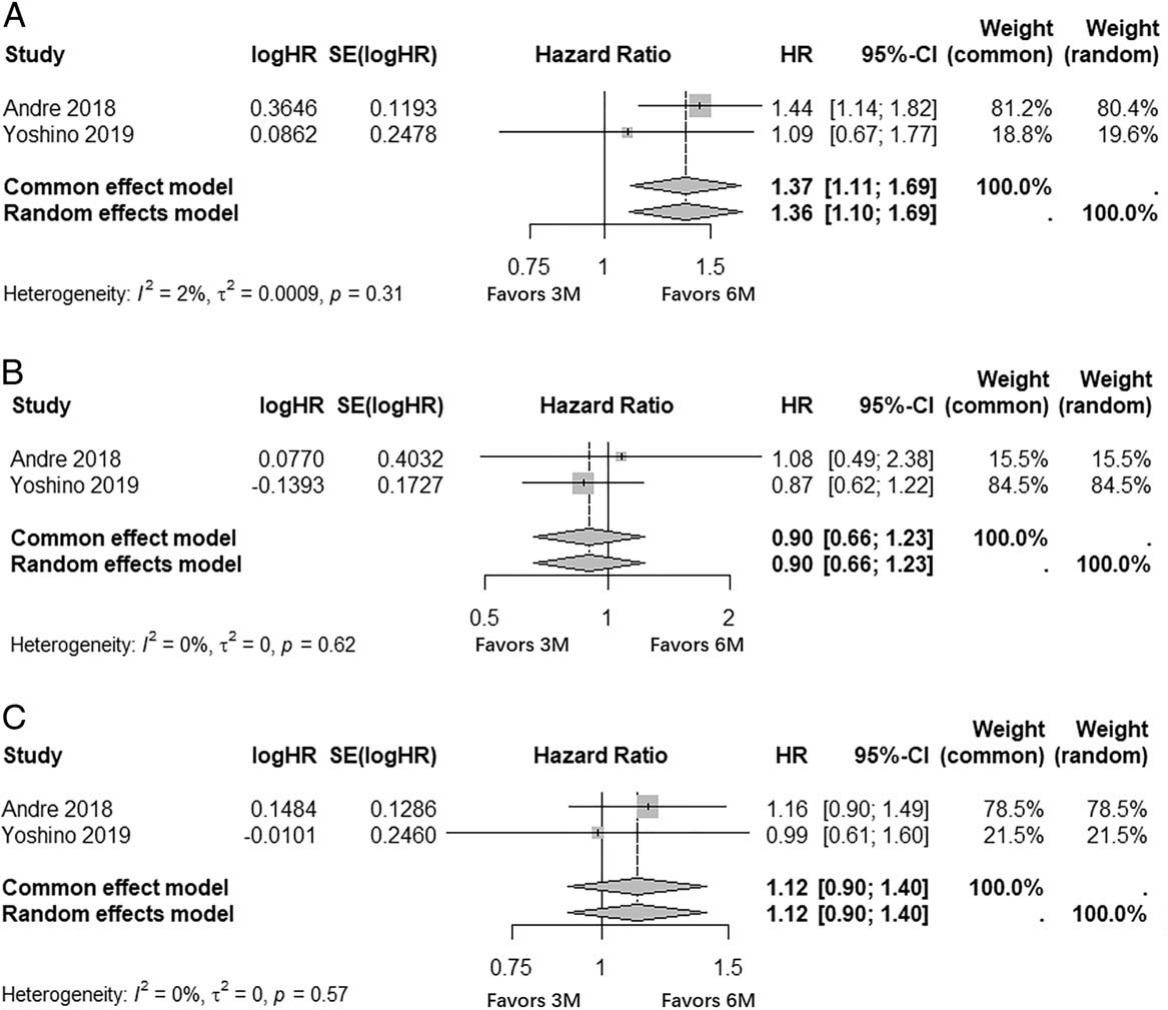
Assessment of survival outcomes between 3-month and 6-month adjuvant chemotherapy in colon cancer patients. (A) Disease-free survival (DFS) between 3-month and 6-month FOLFOX in high-risk stage III patients; (B) overall survival (OS) between 3-month and 6-month CAPEOX in stage III patients; (C) OS between 3-month and 6-month FOLFOX in stage III patients.

Thirdly, Figure 5^1^ erroneously incorporates the data from Kim *et al*.^3^, where the included data pertains to stage II/III rather than stage III colon cancer. Furthermore, it is essential to include another study by Yoshino *et al*.^4^ that meets the inclusion criteria. The reanalyzed pooled outcome reveals no statistically significant difference in overall survival (OS) between 3-month and 6-month CAPEOX regimens in patients with stage III colon cancer (HR=0.90 [0.66–1.23]; *I*^2^=0%; Fig. 1B).

Finally, Yoshino *et al*.^4^ also reported the OS of 3-month vs. 6-month FOLFOX in patients with stage III CC, which should be included in the analysis. The pooled analysis revealed no statistically significant difference in the OS between patients receiving 3-month and 6-month FOLFOX regimens for stage III colon cancer (HR=1.12 [0.90–1.40]; *I*^2^=0%; Fig. 1C).

## Ethical approval

No need for this Letter to the Editor.

## Sources of funding

This work was supported by the Joint project of the Chongqing Health Commission and Science and Technology Bureau (No. 2021MSXM309).

## Author contribution

H.P. and M.Y.: original draft conception and writing; W.L., S.Z., and H.S.: critical revision of the manuscript. All authors reviewed the manuscript.

## Conflicts of interest disclosure

The authors have no conflicts of interest to declare.

## Research registration unique identifying number (UIN)

1. Name of the registry: none.
2. Unique identifying number or registration ID: none.
3. Hyperlink to your specific registration (must be publicly accessible and will be checked): none.

## Guarantor

Hao Sun.

## Provenance and peer review

Commentary, internally reviewed.
